# Imaging of giant cell tumor of bone

**DOI:** 10.4103/0019-5413.32037

**Published:** 2007

**Authors:** Shaligram Purohit, Dinshaw N Pardiwala

**Affiliations:** Department of Orthopaedics, King Edward VII Memorial Hospital, Parel, Mumbai - 400 012, India

**Keywords:** Giant cell tumor, imaging, magnetic resonance imaging

## Abstract

Giant cell tumor (GCT) of bone is a benign but locally aggressive and destructive lesion generally occurring in skeletally mature individuals. Typically involving the epiphysiometaphyseal region of long bones, the most common sites include the distal femur, proximal tibia and distal radius. On radiographs, GCT demonstrates a lytic lesion centered in the epiphysis but involving the metaphysis and extending at least in part to the adjacent articular cortex. Most are eccentric, but become symmetric and centrally located with growth. Most cases show circumscribed borders or so-called geographical destruction with no periosteal reaction unless a pathological fracture is present. There is no mineralized tumor matrix. Giant cell tumor can produce wide-ranging appearances depending on site, complications such as hemorrhage or pathological fracture and after surgical intervention. This review demonstrates a spectrum of these features and describes the imaging characteristics of GCT in conventional radiographs, computerized tomography scans, magnetic resonance imaging, bone scans, positron emission tomography scans and angiography.

Giant cell tumor (GCT) of bone is a benign but locally aggressive and destructive lesion composed of primitive histiocytes and diffuse, large, multinucleated giant cells.

## Epidemiology

In the orient, GCT may account for 20% of all primary skeletal neoplasms.^1^ Generally occurring in skeletally mature individuals with its peak incidence in the third decade of life,^2^ less than 2% are found in patients with open epiphyses.^3^ There is a slight female predominance (56.4% in one large series).^2^ Giant cell tumor of the small bones of the hand and foot seems to occur in a slightly younger age group and demonstrates a higher incidence of multicentricity than in other locations.^4^,^5^

## Skeletal distribution

Almost always a mono-ostotic process, the most common sites include the distal femur, proximal tibia and distal radius. The sacrum, distal tibia, proximal humerus, proximal femur, pelvis and proximal fibula are not infrequent sites. Rarely, bones of the hand and feet, vertebral bodies and ribs may be involved.^2^,^6^,^7^

## Pathology

On gross pathology, GCT typically involves the epiphysiometaphyseal region of long bones. The tumor almost always extends up to the adjacent articular cartilage, which remains intact and, rarely, when neglected, it may involve the diaphysis because it may attain immense size. The tumor is usually eccentric to the long axis of the bone but may be centrally located. Predominant metaphyseal involvement with epiphyseal extension through the growth plate has been noted in a small number of skeletally immature patients.^8^

The overlying cortex has usually undergone resorption and the contour of the bone is expanded by the tumor which is covered by a thin shell of subperiosteal new bone. Areas of necrosis and hemorrhage may result in cystification of the tumor, which may be so prominent as to mimic aneurysmal bone cyst.^2^,^7^,^9^,^10^

## Histopathology

Histologically the lesion is composed of osteoclast-like multinucleated giant cells in a moderately vascularized network of proliferating round, oval or spindle-shaped stromal cells. Ossification and osteoid production are noted in small foci at the periphery of the lesions, particularly in soft tissue extensions.^2^,^7^,^9^–^11^

## Clinical behavior

Giant cell tumors are prone to local recurrence. Although benign, in 3.5% of cases they show metastasis to the lungs and more rarely to other sites, where the secondaries are histologically benign and identical to the primary lesion.^9^,^12^,^13^ Metastasis is more commonly seen from primary sites like the sacrum and radius and may also be related to previous surgical intervention or irradiation on the primary lesion.^14^,^15^

A sarcoma may occur in conjunction with a histologically benign GCT or it may develop at the site of a previously treated GCT after a prolonged interval, usually following irradiation. This occurrence is generally known as a malignant giant cell tumor in which the malignancy is usually an osteosarcoma, a malignant fibrous histiocytoma or a fibrosarcoma.^2^

GCT can produce wide-ranging appearances depending on the site, complications such as hemorrhage or pathological fracture and after surgical intervention. This review demonstrates a spectrum of these features.

## RADIOGRAPHIC FEATURES

The typical giant cell tumor of the epiphysis is a solitary, relentlessly growing neoplasm that results in extensive bone resorption. On rare occasions it may present in multiple bones.

## LONG AND TUBULAR BONES

### Epiphyseal location

**Figure 1:**
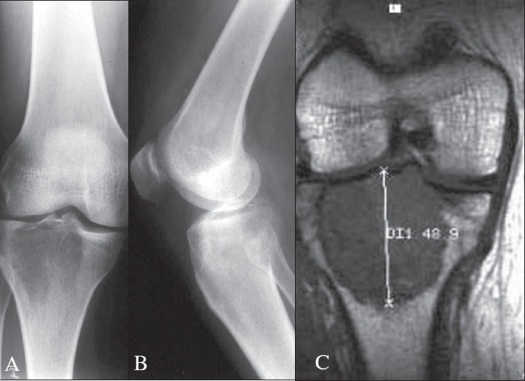
Antero-posterior A) and lateral x-rays. B) with coronal MRI. C) of a GCT of proximal tibia showing a lytic lesion, with sharp, well defined margins and extensive subchondral lysis

GCT demonstrates a lytic lesion centered in the epiphysis but involving the metaphysis and extending at least in part to the adjacent articular cortex [Figure 1]. Less than 2% present in the metaphysis or diaphysis 16 and in such instances the pathologist must prove that the lesion is not a giant cell rich osteosarcoma or a bone lesion of hyperparathyroidism.

### Intramedullary eccentric versus central location

**Figure 2:**
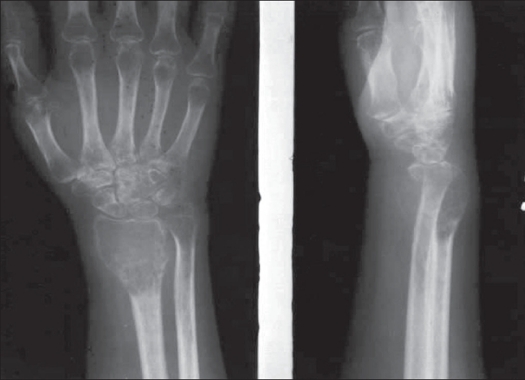
Antero-posterior and lateral x-rays of a GCT of distal radius showing cortical expansion and areas of cortical erosion

In the major long bones such as the femur and tibia, all lesions begin in the intramedullary region. Most are eccentric, but become symmetric and centrally located with growth [Figure 1]. In the thin long bones, such as the fibula or radius, most lesions are centrally placed from initial presentation [Figure 2].

### Geographical destruction

Most cases show circumscribed borders or so-called geographical destruction [Figure 1]. In 10% the edges may appear permeative to moth-eaten.^17^ These differences reflect the variability in the lesion's growth rates. The pathologist must exclude a giant cell rich osteosarcoma or secondary aneurysmal bone cyst engrafted on a GCT in these cases.

### Confinement to bone

**Figure 3:**
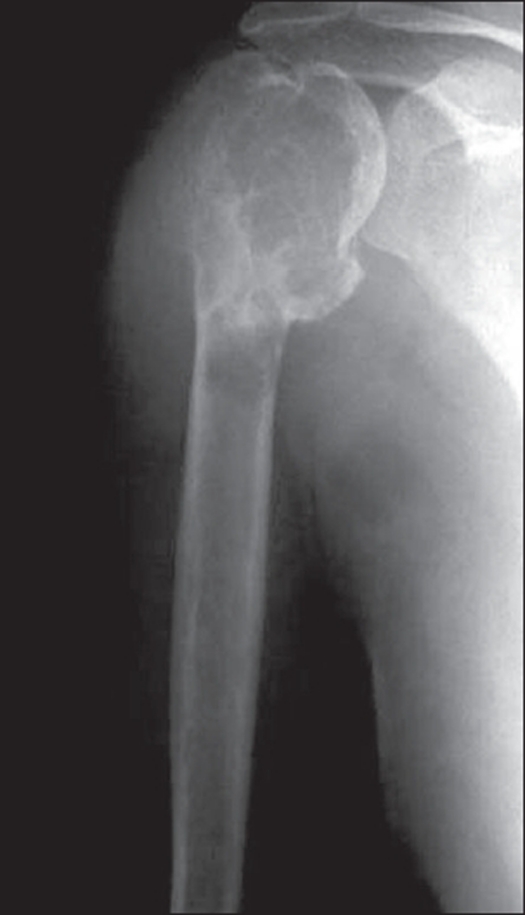
X-rays of GCT of proximal humerus showing extensive cortical destruction.

Early lesions are contained within the original bone contours. With growth, the tumor usually bulges beyond the confines of the cortex, which undergoes varying degrees of resorption. A significant percentage may cause eccentric or concentric cortical erosion and extend into soft tissues [Figure 3].

### Extension to articular cartilage

Most GCT abut a border of the articular cartilage (subchondral bone) in one or more planes [Figures 1 and 2].

### Lysis with and without trabeculation

Lysis is common to all GCT, probably due to massive osteoclastic proliferation. Peripheral bony ridges of a lobulated tumor give the radiographic appearance of trabeculations [Figure 1]. These trabeculations appear as a filigree of coarse to fine honeycomb-like patterns.

### Absence of benign host bone sclerosis

The margins of the lesion bordering the adjacent cancellous bone may be well defined or ill defined and seldom a thin shell of reactive bone may be present. Less than 5% of GCT have a ring of benign host bone sclerosis and these may represent rare, older to regressing forms of GCT.^17^

### Absence of punctate calcifications, intralesional bone formation or periosteal reaction

Apart from a thin shell of subperiosteal new bone outlining the outer surface of the tumor, no periosteal reactions are appreciated unless a pathological fracture is present. There is no mineralized tumor matrix.

### Spine and flat bones

The radiographic features of GCT at sites other than the long bones are nonspecific and not unlike those of other osteolytic processes. Giant cell tumor of the spine almost always begins in the vertebral body and may lead to vertebral collapse or extend into the intervertebral disc, adjacent vertebral body, spinal canal or paraspinal soft tissues. 18 Sternal and sacral lesions are osteolytic and owing to a large size and a soft tissue component, may simulate the appearance of a malignant neoplasm. In the sacrum, the eccentric location and abutting of the SI joint differentiate GCT from similar appearing sacral chordomas. In the sacrum transarticular extension of the tumor may be noted.

### Multicentric GCT

Rarely, two or more bones may be involved by GCT.^19^ In Mirra's series the incidence was 1.3% of conventional GCT.^17^

## COMPUTED TOMOGRAPHY (CT)

**Figure 4:**
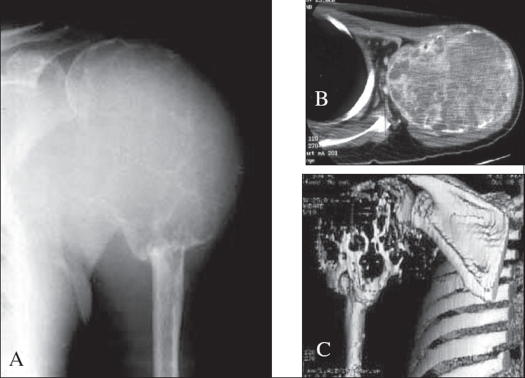
GCT of proximal humerus with extensive cortical expansion and destruction. A) X-rays showing extension to subchondral bone, lysis with trabeculation, and absence of benign host bone sclerosis. B) Axial CT showing multiple areas of cortical erosion. C) 3D reconstruction delineating the spatial extent of the tumour

Plain radiographs remain the mainstay of the diagnosis of GCTs, however, MRI and CT are important for staging and therefore surgical planning. CT will rarely add additional information that changes the differential diagnosis.^20^ However, CT is superior to conventional radiography and tomography in outlining tumor extent [Figure 4], especially its extra-osseous portion and its relationship to adjacent structures, as well as evaluation of cortical integrity and determination of tumor recurrence.^21^,^22^ The expanded and thinned cortex is vividly demonstrated and the presence or absence of matrix calcification can be assessed. Fluid levels may be seen^23^,^24^ secondary to an aneurysmal bone cyst component or due to intratumoral hemorrhage. Reactive changes and edema on the outer cortical surface or the synovium may mimic tumor extension. The axial slices provided by CT do not allow accurate evaluation of the subarticular cortex because of volume averaging.

**Figure 5:**
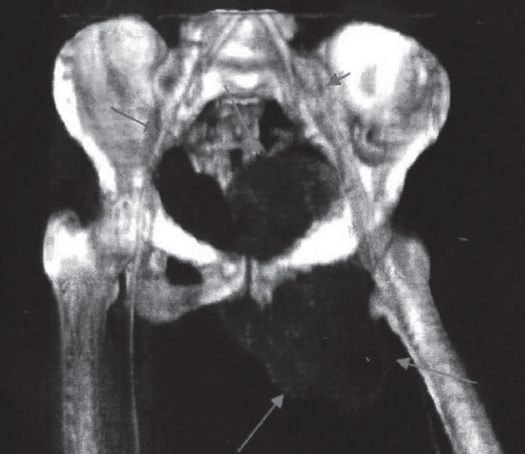
Color volume rendered 3D CT of a left iliac GCT. Note the spatial depiction of the tumor with nearby common iliac vessels

The advent of color volume rendered three-dimensional (3D) CT with video files allows evaluation of multiple tissues at the same time. The spatial depiction of the tumor along with surrounding anatomical relationships such as vessels and ureter make this a useful preoperative imaging modality in cases of pelvic GCT [Figure 5]. Manipulation and rotation of the 3D images through 360 degrees allows the surgeon a better understanding of the extent of the mass and anticipated surgical complexities.

## MAGNETIC RESONANCE IMAGING (MRI)

**Figure 6:**
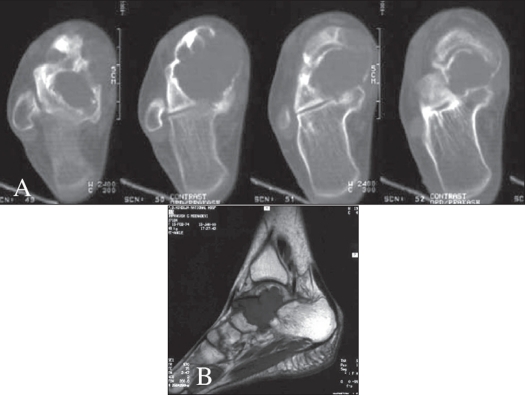
GCT of talus A) CT and B) Sagittal MR shows cortical destruction and extension into soft tissues, subtalar joint and talonavicular joint. The ankle joint is preserved

MRI is currently the best imaging modality for GCT because of its superior contrast resolution and multiplanar imaging capabilities that allow accurate tumor delineation.^20^,^25^ MRI is useful in determining extraosseous extent and articular surface involvement,^26^ however subtle cortical destruction is better demonstrated by CT [Figure 6]. MRI is also useful in assessing intraosseous and intramedullary skip lesions.

GCT shows low intensity on T1 and heterogeneous high intensity on T2 weighted images. Therefore intramedullary tumor is best seen on T1W, while its extraosseous portion is best appreciated on T2W images.^26^,^27^

The hypervascular stroma contains sinusoidal vessels which predisposes to hemorrhage.^8^ The phagocytosed erythrocytes lead to iron deposition in the form of hemosiderin.^28^ Giant cell tumors often have extensive hemosiderin deposition within tumor tissue, resulting in a very low signal intensity on all pulse sequences.^28^ This is seen in up to 60% of cases.^28^ Low signal areas may also be due to collagen deposition secondary to surgery or trauma.^28^ Gadolinium enhancement reveals areas of hypervascularity and enhancement with a very heterogeneous signal pattern.^29^

## RADIONUCLIDE SCINTIGRAPHY (BONE SCAN)

GCT produces increased uptake of technitium-99m radiopharmaceuticals. The pattern of increased uptake may be diffuse (40%) or peripheral with little central activity (60%).^30^ Extended patterns of radioactivity uptake beyond the margins of the tumor preclude accurate definition of intramedullary extent.^21^ Increased uptake in the bone across the adjacent joint and in other joints of the same extremity not involved by tumor may occur.^31^ Therefore the role of bone scan in GCT is limited because it is nonspecific and unreliable in defining the extent of the tumor.^30^ It is however, helpful in evaluating the rare patient with multicentric or metastatic GCT.

## ANGIOGRAPHY

Although angiography is seldom used as a diagnostic modality in the era of CT and MRI, it can determine the extra-osseous extent of the tumor and its relationship to major vessels. The majority of GCT are hypervascular, but 10% aneurysmal bone cyst components may be completely avascular. Reactive hyperemic synovium may mimic extraosseous tumor extension.^27^

The role of angiography today, in patients with GCT, is limited to a study of regional vascular anatomy and perhaps, preoperative transcatheter arterial embolization to facilitate excision and decrease surgical blood loss or in instances of unresectable neoplasms.^32^

### The role of embolization for unresectable tumors

Unresectable GCTs (e.g., certain sacral and pelvic tumors) can be managed with transcatheter embolization of their blood supply. Since flow reconstitution invariably occurs, embolization is performed at monthly intervals until significant pain palliation is achieved. Subsequent embolizations are performed when there is symptomatic or radiographic relapse of the tumor.

## POSITRON EMISSION TOMOGRAPHY (PET) SCAN

PET allows the visualization of the metabolic activity of disease. In orthopedic surgery it is of utmost help in the diagnosis of malignant tumors and their recurrence, the staging of tumors and the monitoring of their response to therapy. Although the role of PET in GCT is as yet to be defined, this imaging modality holds great promise. Definition of the primary tumor with a number of radiotracers will allow the determination of blood flow, the turnover of DNA, the turnover of amino-acids, hypoxia of the tumor and the glucose metabolism. This will enable metabolic staging of the tumor, which may have a predictive value equal to or surpassing histological techniques.^33^

## EVALUATION OF LOCAL RECURRENCE

**Figure 7:**
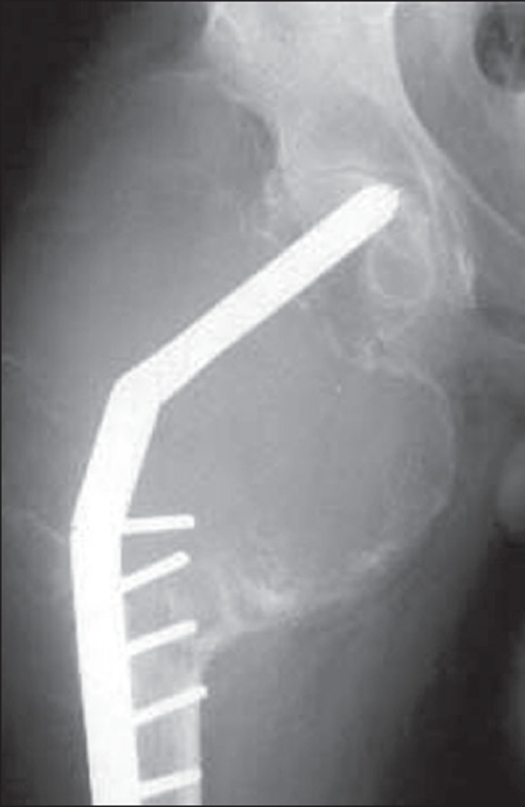
A case of recurrent GCT of proximal femur at 18 months follow up. The x-rays shows extensive cortical expansion with impending implant breakage

**Figure 8:**
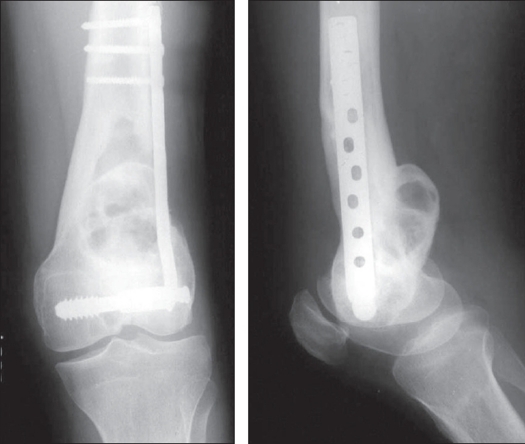
Recurrent GCT of the distal femur in a patient having undergone intralesional curettage, bone grafting and fixation. Note the intraosseous geographical destruction with cortical expansion

Local recurrences manifest themselves within three years in 80-90% of cases^6^ and appear to be related to the surgical margin.^6^ Clinically characterized by pain, the radiographic features include:

1. Progressive lysis of the bone graft, which may have become incorporated or in the adjacent cancellous bone, in patients having undergone intralesional curettage with bone grafting [Figures 7 and 8].
2. Following curettage and cementation an osteolytic zone caused by thermal injury measuring 2 mm surrounds the cement. This radiolucent zone is bordered by a thin outer sclerotic rim for about six months.^14^,^34^ Progressive lysis or failed development of the sclerotic rim between the cement and cancellous bone suggests recurrence.^34^,^35^
3. Although recurrence usually occurs in the parent bone, soft tissue implantation can occur at the time of surgery and may be the only site of disease. Soft tissue recurrence is visible on plain radiographs because of its tendency towards peripheral calcification.^11^

MRI is the optimum technique for evaluation of recurrent or residual disease. Local postoperative high signal within the surgical bed that exhibits a rounded mass-like appearance with eccentric growth is highly suggestive of tumor.^36^ Differentiation of recurrent tumor from cement-related giant cell reaction can sometimes be difficult. Giant cell granulomas usually develop after several years while the majority of tumor recurrences occur within 18 months after the initial surgery. In addition, tumor recurrence grows more rapidly than giant cell granuloma. However, overlap of features between these two entities can occur and a CT-guided core biopsy may be needed.
